# Developing a Gene Biomarker at the Tipping Point of Adaptive and Adverse Responses in Human Bronchial Epithelial Cells

**DOI:** 10.1371/journal.pone.0155875

**Published:** 2016-05-19

**Authors:** Jenna M. Currier, Wan-Yun Cheng, Daniel Menendez, Rory Conolly, Brian N. Chorley

**Affiliations:** 1 Oak Ridge Institute for Science and Education at U.S. Environmental Protection Agency, Research Triangle Park, North Carolina, United States of America; 2 Genome Integrity & Structural Biology Laboratory, National Institute of Environmental Health Sciences, NIH, Research Triangle Park, North Carolina, United States of America; 3 National Health and Environmental Effects Research Laboratory, U.S. Environmental Protection Agency, Research Triangle Park, North Carolina, United States of America; Roswell Park Cancer Institute, UNITED STATES

## Abstract

Determining mechanism-based biomarkers that distinguish adaptive and adverse cellular processes is critical to understanding the health effects of environmental exposures. Shifting from *in vivo*, low-throughput toxicity studies to high-throughput screening (HTS) paradigms and risk assessment based on *in vitro* and *in silico* testing requires utilizing toxicity pathway information to distinguish adverse outcomes from recoverable adaptive events. Little work has focused on oxidative stresses in human airway for the purposes of predicting adverse responses. We hypothesize that early gene expression-mediated molecular changes could be used to delineate adaptive and adverse responses to environmentally-based perturbations. Here, we examined cellular responses of the tracheobronchial airway to zinc (Zn) exposure, a model oxidant. Airway derived BEAS-2B cells exposed to 2–10 μM Zn^2+^ elicited concentration- and time-dependent cytotoxicity. Normal, adaptive, and cytotoxic Zn^2+^ exposure conditions were determined with traditional apical endpoints, and differences in global gene expression around the tipping point of the responses were used to delineate underlying molecular mechanisms. Bioinformatic analyses of differentially expressed genes indicate early enrichment of stress signaling pathways, including those mediated by the transcription factors p53 and NRF2. After 4 h, 154 genes were differentially expressed (*p* < 0.01) between the adaptive and cytotoxic Zn^2+^ concentrations. Nearly 40% of the biomarker genes were related to the p53 signaling pathway with 30 genes identified as likely direct targets using a database of p53 ChIP-seq studies. Despite similar p53 activation profiles, these data revealed widespread dampening of p53 and NRF2-related genes as early as 4 h after exposure at higher, unrecoverable Zn^2+^ exposures. Thus, in our model early increased activation of stress response pathways indicated a recoverable adaptive event. Overall, this study highlights the importance of characterizing molecular mechanisms around the tipping point of adverse responses to better inform HTS paradigms.

## Introduction

In the years since the National Research Council’s 2007 report, “Toxicity Testing in the 21st Century: A Vision and a Strategy,” significant effort has focused on implementing *in vitro* and *in silico* predictive toxicity testing as opposed to expensive, low throughput *in vivo* studies [1]. For risk-based prioritization and regulatory decision making, high-throughput screening (HTS) assays examine the cellular and molecular pathways that mediate adverse effects when sufficiently perturbed in response to chemical exposure. Several HTS paradigms and risk assessment frameworks are well underway, including the interagency agreement, Toxicity Testing in the 21^st^ Century (Tox21) [2, 3] and the U.S. Environmental Protection Agency’s Toxicity Forecaster (ToxCast) program [4, 5]. Although considerable progress has been made on the technical and theoretical challenges facing HTS, implementation to predict adverse human outcomes while minimizing or eliminating the use of *in vivo* studies has proved difficult [6]. Challenges arise in the interpretation of *in vitro* results and utilizing toxicity pathway information to distinguish adverse outcomes from recoverable adaptive events.

Simmons et al. (2009) suggested focusing on limited number of stress pathways common to most cell types that regulate homeostasis and ultimately determine cell fate decisions through the analysis of transcription factor activation by reporter gene assays [7]. The toxicity associated with chemical stressors likely arises when perturbations sufficiently overwhelm these adaptive stress response pathways [8]. For chemical exposures eliciting oxidative stress, current HTS paradigms measure activation of the stress pathway-related transcription factors commonly induced target genes at limited time points. More recently, high-content imaging (HCI) strategies [9, 10] have been used for dynamic pathway and endpoint assessment of single cells to assess the tipping points between adaptive and adverse phenotypic outcomes. Additionally, a study investigating the adverse effects of cigarette smoke components revealed that whole-genome transcriptomics identified toxicity mechanisms at lower doses and earlier time points compared with high-content screening, indicating that gene expression profiling is useful for the early prediction of latter adverse effects [11]. Interestingly, Ludwig et al. (2011) established a point of departure for gene expression changes for testicular toxicity *in vivo* 5-fold lower than typical toxicological endpoints collected at the same time point [12]. McMillian et al. (2004) developed a 64 gene set signature to identify oxidant exposures eliciting hepatotoxicity *in vivo* [13]. Furthermore, Ryan et al. (2016) established a set of biomarker genes capable of predicting activation of the estrogen receptor α (ERα) using breast cancer-derived cell line MCF-7, which is perturbed by endocrine disrupting chemicals [14]. Cumulatively, the published evidence suggests that early gene expression-mediated molecular changes can be used to delineate adaptive and adverse responses to chemical exposures.

Here, we characterized oxidative stress responses of the tracheobronchial airway to a model oxidant, zinc (Zn), and explored the genomic mechanisms mediating the switch from recoverable adaptive cellular response to adverse events. Zn, a component of airborne particulate matter (PM), presents an oxidant challenge to human lung [15]. Several *in vivo* rodent [16–19] and human epidemiological [20–24] studies have implicated the presence of Zn in PM as mediating adverse pulmonary and cardiovascular outcomes. As an essential element critical to normal cellular function, intracellular Zn is tightly buffered through protein binding and vesicle sequestration. While total cellular Zn concentration in mammalian cells ranges from 100–500 μM, most is tightly bound and unavailable for use in cellular reactions with only femto- or nanomolar quantities in the form of free cytosolic Zn^2+^ [25]. Saturation of intracellular Zn homeostasis resulting in increased free cytosolic Zn^2+^ mediates the adverse oxidative effects of this transition metal [26]. For example, Zn^2+^ translocates to the mitochondria and induces ROS production through inhibition of cytochrome C oxidase [27, 28]. Increases in intracellular free Zn^2+^ *in vitro* have resulted in the activation of stress signaling pathways and p53-mediated apoptosis [29]. Moreover, redox imbalance and the induction of adaptive and inflammatory gene expression were observed in bronchial epithelial cell cultures exposure to Zn^2+^ [30].

To explore the molecular mechanisms mediating the shift from adaptive to adverse responses in human airway in response to oxidative insult, we first determined normal, adaptive, and cytotoxic Zn^2+^ exposure conditions in a normal human bronchial epithelial cell line (BEAS-2B). Global gene expression measurements under these conditions were conducted to delineate underlying molecular mechanisms. We then identified candidate genes for use as early biomarkers in differentiating adaptive and adverse cellular responses.

## Materials and Methods

### Cell culture and treatment

The SV-40 transformed human bronchial epithelial cell line, BEAS-2B, was obtained from American Type Culture Collection (Manassas, VA) and were cultured in supplemented serum-free keratinocyte growth medium (KGM-Gold, Lonza Ltd., Basel, Switzerland). Cells from passage numbers 48–60 were seeded in to tissue culture treated plates and flasks (Corning, Inc., Corning, NY) manufactured within the last two years, which produced consistent monolayer cultures, and maintained at 37°C in a humidified incubator with a 95% air/5% CO_2_ atmosphere. Prior to treatment, cells were starved overnight in serum-free keratinocyte basal medium (KBM-Gold, Lonza Ltd.). To elicit adaptive and adverse cellular responses, cultures were exposed to Zn^2+^ as zinc sulfate heptahydrate (ZnSO_4_∙7H_2_0, ≥99%) and 1 μM of 2-mercaptopyridine N-oxide sodium salt (pyrithione, ≥96%), a Zn specific ionophore to facilitate Zn^2+^ transport, obtained from Sigma-Aldrich (St. Louis, MO).

### Cytotoxicity and glutathione Assays

The luminescent cell viability assay measuring ATP content, CellTiter-Glo, was used to determine non-cytotoxic Zn^2+^ exposures in BEAS-2B cells (Promega, Madison, WI). Zn^2+^-mediated oxidative stress in BEAS-2B cells was assessed by determining intracellular reduced (GSH) and oxidized (GSSG) glutathione levels and with a luciferase-based reporter assay (GSH/GSSG-GLO, Promega), which measures GSSG, total glutathione (GSH + GSSG), and GSH/GSSG ratios. Luminescence was measured with a CLARIOstar microplate reader (BMG Labtech, Inc., Cary, NC) and compared with untreated cells in wells containing KBM-Gold medium. The MTT assay was performed per the manufacturer’s instructions using the Vybrant MTT Cell Proliferation Assay Kit (Life Technologies, Inc., Gaithersburg, MD). Absorbance of solubilized formazan product was quantified with a SpectraMax 340 microplate reader at 570 nm using a reference wavelength of 630 nm (Molecular Devices, Sunnyvale, CA).

### NRF2 activation assays

Nuclear Factor Erythroid 2-Like 2 (NFE2L2 or NRF2) activation and nuclear translocation was determined using an ELISA-based sequence binding assay, the TransAM NRF2 Transcription Factor Activation Kit (Active Motif, Carlsbad, CA). Briefly, nuclear extracts (7 μg) isolated with the Nuclear Extract Kit (Active Motif) were incubated for 1 h in a 96-well plate containing immobilized oligonucleotides containing the antioxidant response element (ARE) consensus binding site. The plate was then washed and incubated for 1 h with a primary antibody (1:1000), specific for an epitope on activated NRF2 protein when bound to DNA. The plate was washed again and incubated for 1 h with horseradish peroxidase-conjugated secondary antibody (1:1000). Absorbance of the colorimetric readout was quantified with a SpectraMax 340 microplate reader at 450 nm.

### Measurement of apoptotic markers: Bead- and capillary nano-immunoassays

Whole cell extracts from Zn^2+^-exposed BEAS-2B cultures, isolated using the Nuclear Extract Kit (Active Motif), were collected and stored at -80°C or used immediately. The following markers of apoptosis were probed using magnetic multiplex bead-based immunoassays (Bio-Rad, Hercules, CA): active caspase-3, Mcl-1/Bak, Bcl-xL/Bax, and survivin. The assay was performed according to the manufacturer’s instructions. Briefly, whole cell extracts were incubated with magnetic capture beads containing primary antibodies of interest in a 96-well plate. Samples were then incubated with biotinylated secondary antibodies and detected with streptavidin-PE using the Bio-Plex MAGPIX reader and Bio-Plex Manager software (Bio-Rad).

Automated capillary nano-immunoassays using the WES instrument (Protein Simple, San Jose, CA) were utilized to detect PARP-1, cleaved PARP, p53, phosphorylated p53 (Ser 20 and Ser 15), and α-Tubulin. All reagents and samples were prepared according to the manufacturer’s protocol for the 12–230 kDa master kit containing cartridges with 25-capillaries. BEAS-2B whole cell extracts were diluted to antibody specific optimized protein concentrations of 0.005–0.6 μg/μL in sample buffer containing master mix. Samples were boiled for 5 min at 95°C, and then transferred to the appropriate wells. The following primary antibodies were obtained from Santa Cruz Biotechnology, Inc. (Dallas, TX): PARP-1 (sc-7150), cleaved PARP (sc-23461-R), and total p53 (sc-126). Phoshorylated-p53 (Ser 15, cat #9286 and Ser 20, cat #9287) and α-Tubulin (cat #3873), used as a loading control, were purchased from Cell Signaling Technology (Boston, MA). Goat anti-mouse and anti-rabbit HRP conjugate secondary antibodies were obtained from Protein Simple. Protein separation by electrophoresis and immunodetection were carried out at room temperature in the automated capillary cartridge system and data were analyzed with the Compass software (Protein Simple). Differences in α-Tubulin normalized peak areas from three independent experiments are presented as fold over control.

### RNA extraction

Total RNA was isolated from Zn^2+^-exposed BEAS-2B cells using RNAzol®RT (Molecular Research Center, Cincinnati, OH) and purified with RNeasy MinElute columns (Qiagen GmbH, Hilden, Germany). RNA was quantified using the NanoDrop spectrophotometer (NanoDrop Technologies, Wilmington, DE) and evaluated for integrity with an Agilent 2100 Bioanalyzer (Agilent Technologies GmbH, Berlin, Germany).

### Microarray analysis

To assess global gene expression, samples were hybridized onto HumanHT-12 V4.0 Expression BeadChip arrays (Illumina, San Diego, CA) in the NHEERL Genomic Research Core Laboratory using standard Illumina protocols. Arrays were scanned and raw data (.idat files) were obtained using Illumina iScan software (v3.3.28) and analyzed with Illumina GenomeStudio® Data Analysis Software. Array data is publicly available at Gene Expression Omnibus, accession number GSE80733. Raw gene expression intensities were quantile normalized using Illumina GenomeStudio® and imported in to Partek Genomics Suite (Partek, St. Louis, MO). The following parameters were determined for each probe: (i) the fold-change in gene expression between exposed and control samples and (ii) Student’s t test comparing adaptive and adverse Zn^2+^ exposures (*p* <0.01). An ANOVA was conducted to determine differentially expressed genes and probes were filtered based on (i) fold-change in either direction ≥2.0 and (ii) *q*-value <0.05 (False Discovery Rate-adjusted *p*-value). Ingenuity Pathway Analysis software (IPA, Qiagen, Redwood City) was used to map differentially expressed genes to canonical pathways and to visualize biological networks.

A commercially available gene expression database (NextBio, Illumina; http://www.nextbio.com) was used to compare gene expression results to other studies [31]. Biosets generated from experiments using human bronchial tissues or cells were used in the analysis. Gene sets were imported into NextBio and individual genes were rank-ordered based on their fold-change. The sets were then compared to publically available biosets in the NextBio database using a pair-wise rank-based algorithm (the Running Fisher test).

### Quantitative real-time polymerase chain reaction (RT-qPCR) analysis

Total RNA was isolated as previously described and then exposed to DNAse prior to purification with RNeasy MinElute columns (Qiagen). Next, cDNA was synthesized with iScript Reverse Transcription Supermix (Bio-Rad) from up to 1 μg of total RNA. RT-qPCR was carried out using a Bio-Rad CFX384 Real-Time PCR Detector with SsoFast EvaGreen Supermix, 8 ng cDNA, and gene specific PrimePCR SYBR Green Assays.

### Comparison with published p53 and NRF2 ChIP-seq data

Previously published p53 ChIP-seq, ChIP-exo and GRO-seq studies, and related gene expression data, were retrieved from the NCBI Gene Expression Omnibus for the following studies and its supplemental materials [32–39]. The studies were performed in six different human cell lines (HCT116, U20S, MCF7, CAL51 and SaOS2) and 8 different conditions (untreated, Doxorubicin, Nutlin, 5FU, RITA, UV IR and p53 overexpression). For NRF2 analysis, ChIP-seq and gene expression analysis from sulforaphane-induced human lymphoblastoid cells was retrieved from Chorley et al. 2012 and its supplemental materials [40]. Data from these studies was used for comparison with the biomarker genes determined after the Zn^2+^ exposure identified in our study.

### Statistical Analysis

Statistical analysis of data from cell-based assays were performed using SigmaPlot 13 (Systat Software Inc., San Jose, CA). ANOVA, followed by Holm-Sidak post hoc test was used to determine significant differences between control and treated samples. Statistical significance was considered at the level of *p* <0.01 unless otherwise noted. For RT-qPCR analysis, calibrated normalized relative quantities (CNRQ) of target genes were calculated using qBASE software (Biogazelle, Zwijnaarde, Belgium). *HMBS* and *ACTB* were used as endogenous reference genes, based on geNORM selection [41]. Determination of fold-change in gene expression between exposed and control samples and Student’s t test comparing adaptive and adverse Zn^2+^ exposures were performed in Partek Genomics Suite using log normalized CNRQ values.

## Results

### Zn^2+^-mediated cytotoxicity and apoptosis

**Fig 1:**
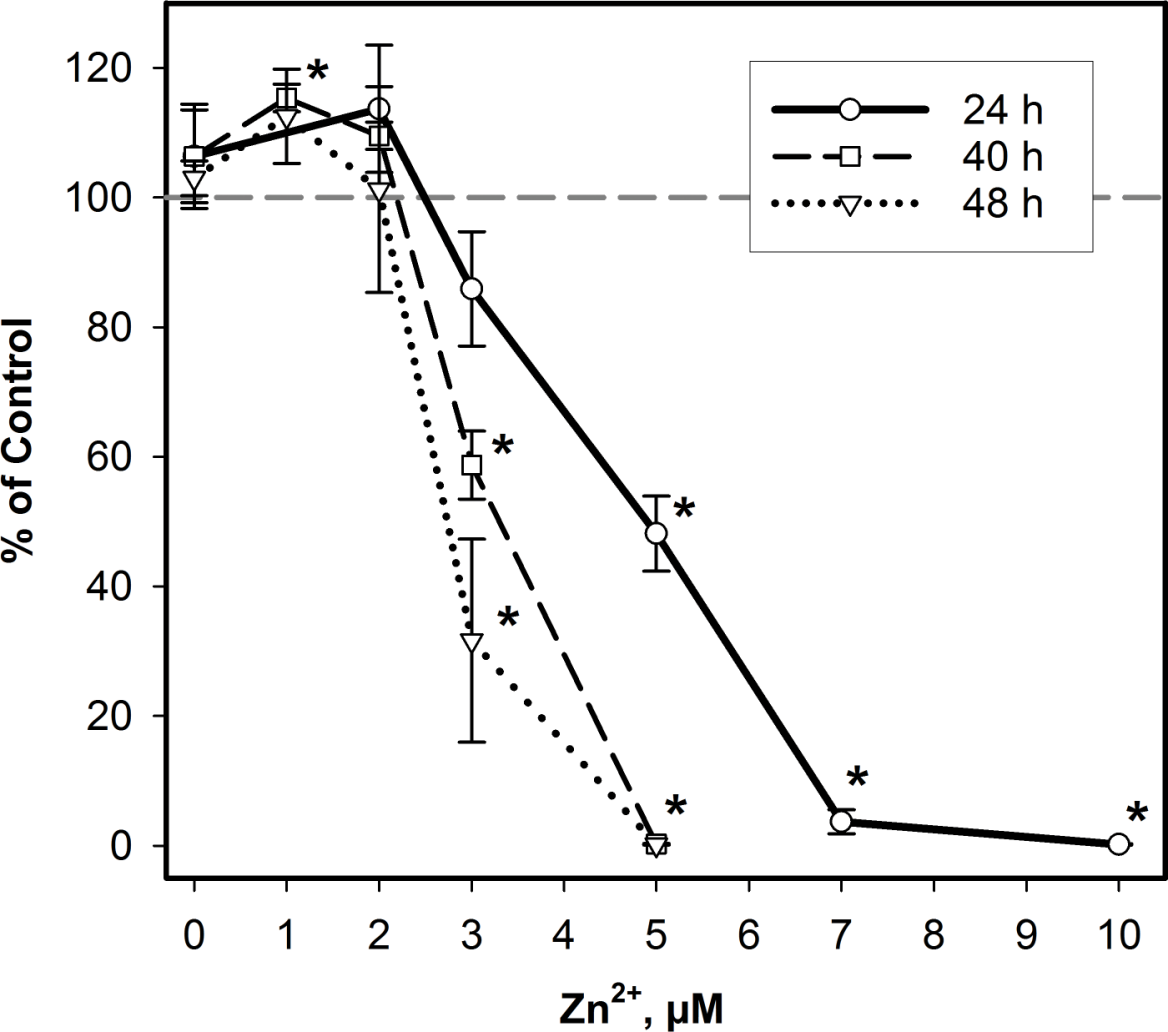
Time- and concentration-dependent cytotoxicity after Zn^2+^ exposure in BEAS-2B cells. A luminescent cell viability assay was used to measure cytotoxicity in BEAS-2B cells exposed to 0–10 μM Zn^2+^ and 1 μM pyrithione for up to 48 h. Results are presented as percent of unexposed control wells (*n* = 3, mean ± SD). (*) *p* <0.01 compared with control by one-way ANOVA followed by Holm-Sidak post hoc test.

BEAS-2B cell cultures were exposed in the presence of Zn specific ionophore, 1 μM pyrithione, for up to 48 h, and ATP levels were measured to assess cellular viability (Fig 1). After 24 h, we observed significantly reduced viability in cells exposed to 5, 7, and 10 μM Zn^2+^ (48±6, 4±2, and 0.2±0.1% viability, respectively) but not in cells exposed to 2 or 3 μM Zn^2+^ or pyrithione alone (114±10, 86±9, and 106±7% viability, respectively). However, cell viability was reduced with 3 μM Zn^2+^ exposure, but not 2 μM or lower, after 40 and 48 h. Interestingly, exposure to 1 μM Zn^2+^ for 40 h significantly increased viability to 115±2% compared with control suggesting enhanced cellular proliferation. Cellular viability assessed by MTT assay after exposure to 0–10 μM Zn^2+^ in the presence of 1 μM pyrithione after 16, 24, and 40 h are in agreement with these data (S1 Fig). Overall, these data indicate that BEAS-2B exposed to at least 3 μM Zn^2+^ for longer than 24 h elicits unrecoverable cell loss, while at lower concentrations, cells either benefited (1 μM Zn^2+^) or adapted (2 μM Zn^2+^) to exposure.

**Fig 2:**
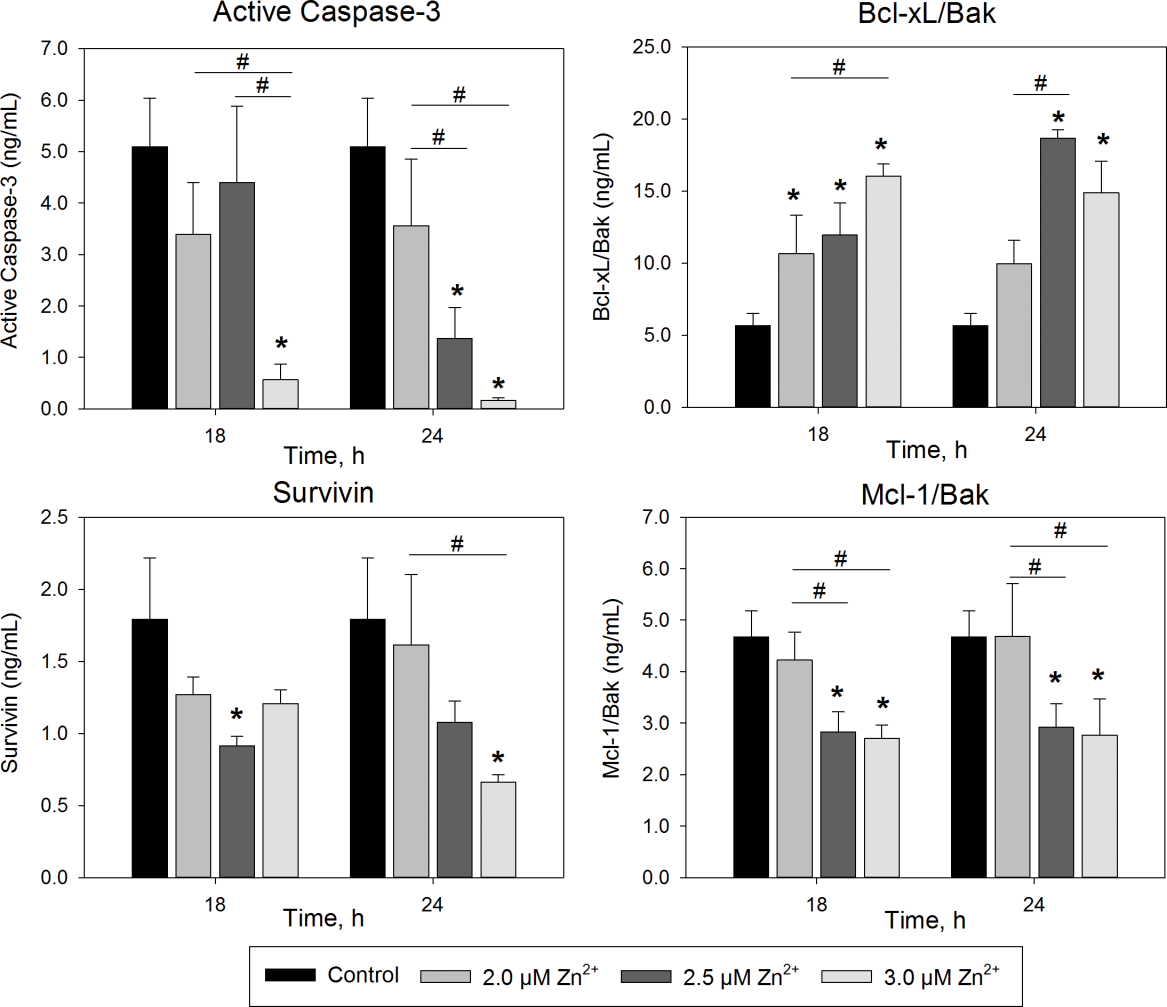
Zn^2+^ exposure in BEAS-2B cells alters protein markers of apoptosis. Whole cell extracts of BEAS-2B cells exposed to 0–3 μM Zn^2+^ and 1 μM pyrithione for up to 24 h were analyzed for protein using a bead-based multiplex immunoassay (*n* = 3, mean ± SD). Significant difference (*p* <0.01) compared with control (*) or each other (#) by two-way ANOVA followed by Holm-Sidak post hoc test.

To determine if the cell loss was due to an apoptotic response, BEAS-2B cells were exposed near the cytotoxic tipping point, at 2, 2.5, and 3.0 μM Zn^2+^ for 18 and 24 h. Both canonical and non-canonical markers of apoptosis were measured, including activated caspase-3 and reduced anti-apoptotic mediators, Mcl-1/Bak, Bcl-xL/Bak, and survivin (Fig 2). Zn^2+^ is a known caspase inhibitor [42, 43] and, not surprisingly, exposure significantly reduced cleavage of caspase-3 in a concentration and time-dependent manner. More reflective of an apoptotic response, survivin levels significantly decreased with 2.5 and 3 μM Zn^2+^ exposure after 18 or 24 h, respectively. In addition, levels of the Mcl-1/Bak complex, which prevents localization of pro-apoptotic Bak to the mitochondrial membrane, also significantly decreased with exposure to 2.5 and 3 μM Zn^2+^. Conversely, Bcl-xL/Bak levels significantly increased with all doses of Zn^2+^ tested at 18h, and only 2.5 and 3 μM after 24 h exposure. With the exception of Bcl-xL/Bak, exposure to 2 μM Zn^2+^ did not significantly alter the apoptotic markers after 18 or 24 h. Overall, these data suggest that apoptotic signaling is perturbed with doses of 2.5 μM Zn^2+^ or greater.

**Fig 3:**
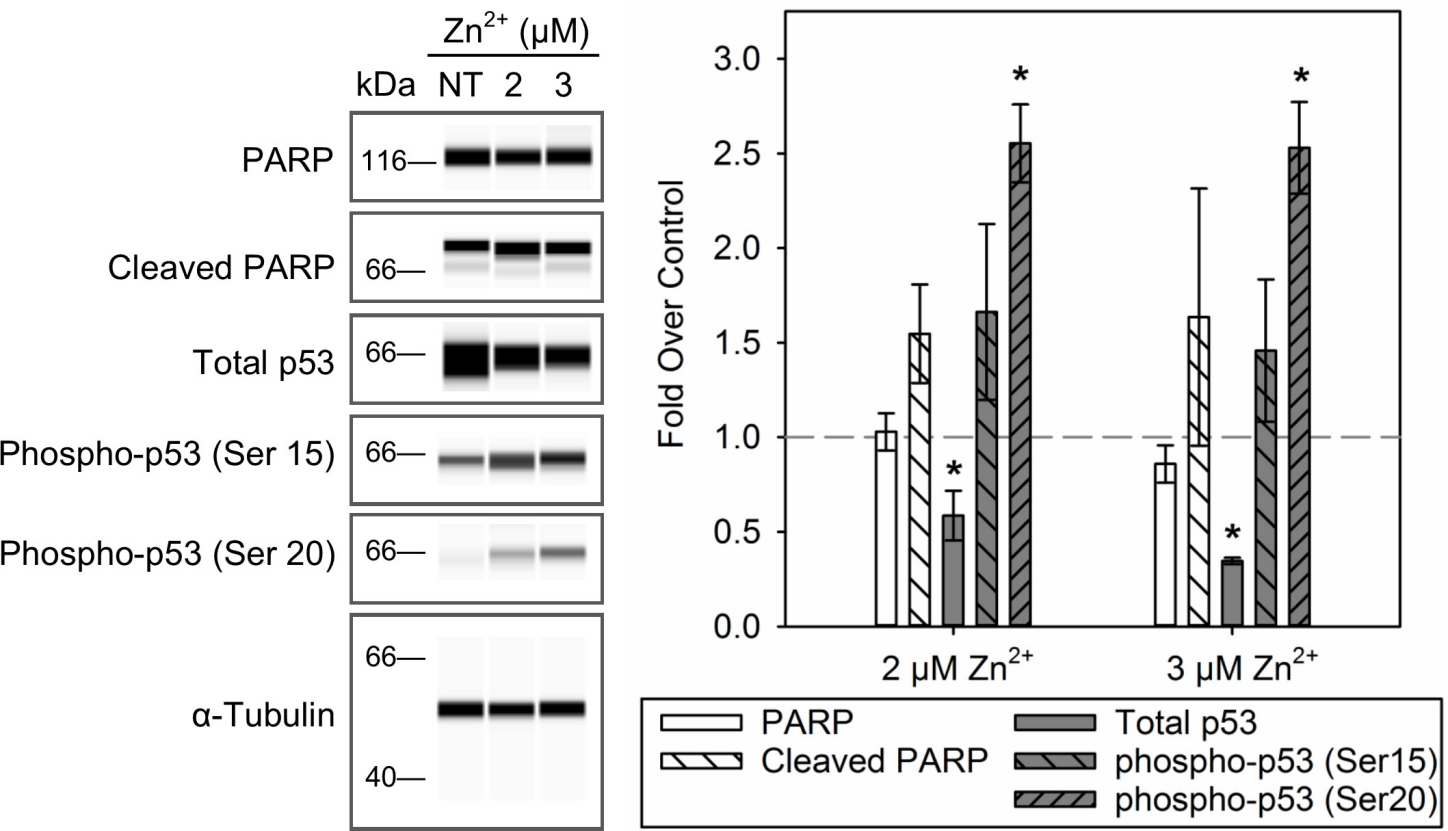
Activation of p53 after Zn^2+^ exposure in BEAS-2B cells. Whole cell extracts from BEAS-2B cells exposed to 2 and 3 μM of Zn^2+^ in the presence 1 μM pyrithione for 4 h were probed for markers of p53 activation and apoptosis using a capillary electrophoresis-based western system. Images (left) are representative rendered blots based on electropherograms of three independent experiments. The chart depicts fold change over control of α-tubulin normalized peak areas for each probed marker (*n* = 3, mean ± SD). Significant difference (*p* <0.05) compared with control (*) by two-way ANOVA followed by Holm-Sidak post hoc test.

To further clarify the timing and mechanisms of these responses, we measured earlier mechanistic markers of apoptosis, activation of p53 and PARP cleavage, after 4 h exposure to 2 and 3 μM Zn^2+^ (Fig 3). Decreased levels of total p53 and increased levels of p53 phosphorylation, a marker of activated p53, were observed. The levels of total p53 were significantly decreased to 0.6±0.1 and 0.35±0.02 fold under control for cells exposed to 2 and 3 μM Zn^2+^, respectively. Slight, but not significant, increases in phospho-p53 (Ser 15) were observed. Moreover, levels of phospho-p53 (Ser 20) were significantly increased to 2.6±0.2 and 2.5±0.2 fold over control, respectively. Exposure to 2 and 3 μM Zn^2+^ for 4 h did not significantly alter the level of PARP in BEAS-2B cells; however, levels of cleaved PARP were slightly increased to 1.7±0.5 and 1.5±0.4 fold over control, respectively. These data suggested that, in our model, p53 is activated early in the course of Zn^2+^ exposures; however, there was no obvious distinction between our adaptive and cytotoxic doses of Zn^2+^ exposure.

### Concentration and duration of Zn^2+^ exposure mediate differential gene expression changes

**Fig 4:**
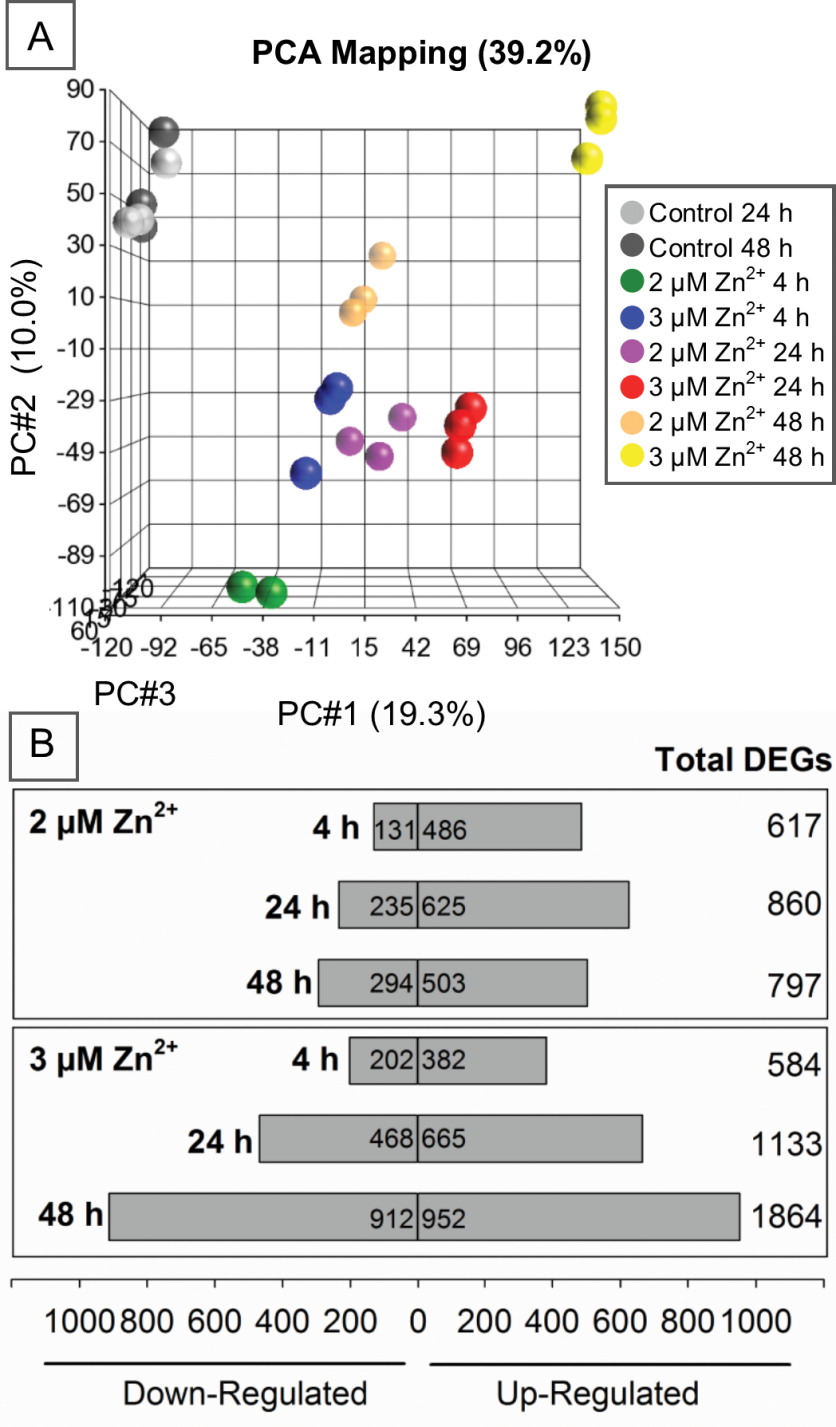
Significant gene expression changes in BEAS-2B cells after Zn^2+^ exposure. Principal Component Analysis (PCA) plot (A) depicting transcriptome differences between microarray data of BEAS-2B cells exposed to up to 3 μM of Zn^2+^ for 4, 24, and 48 h. The number and direction of differentially expressed genes (B) for each treatment group compared with control (n = 3, except n = 2 for 2 μM 4 h exposure group).

Because we observed a clear switch from adaptive to cytotoxic cellular effects between 2 and 3 μM Zn^2+^ doses, we wished to determine if early alterations in gene expression could distinguish this transition. We therefore measured differences in global gene expression under normal, adaptive, and cytotoxic conditions. In total, the expression of 2,588 genes was significantly altered in at least one exposure group. Principal Component Analysis (PCA) clearly discriminated unexposed controls from Zn^2+^-exposed samples (Fig 4A), with minimal variance observed within Zn^2+^-exposed groups. In addition, Zn^2+^-exposed sample groups clustered based on both dose and length of exposure. Sample group distinction was emphasized by the number of non-overlapping genes seen within the same dose group over time, or with different doses of the same exposure length (S2 Fig). Here, 794 genes were differentially expressed under adaptive and/or cytotoxic conditions after only 4 h with approximately 26% and 22% unique to either condition, respectively. Not surprisingly, the 3 μM Zn^2+^ cytotoxic dose at 24 and 48 h elicited the largest and most unique differentially expressed genes (DEGs) when compared to control (Fig 4B). Gene pathway analysis demonstrated that many of the same pathways were altered by both doses, however the levels of enrichment differed (S3 Fig). For example, the significance of enrichment for *Aldosterone Signaling in Epithelial Cells*, *Unfolded Protein Response*, and *NRF2-Mediated Oxidative Stress Response* in cells exposed to 2 μM Zn^2+^ decreased with time, whereas the enrichment was sustained with 3 μM Zn^2+^ exposure. Moreover, the *p53 Signaling* pathway was enriched in 2 and 3 μM Zn^2+^ exposed cells, although similar to the p53 activation studies, no clear distinction in activation or inhibition of this pathway was observed. Overall, these data indicate the activation of several stress related pathways with Zn exposure in BEAS-2B cells.

### Selection of a biomarker gene set differentiating adaptive and cytotoxic cellular responses

**Fig 5:**
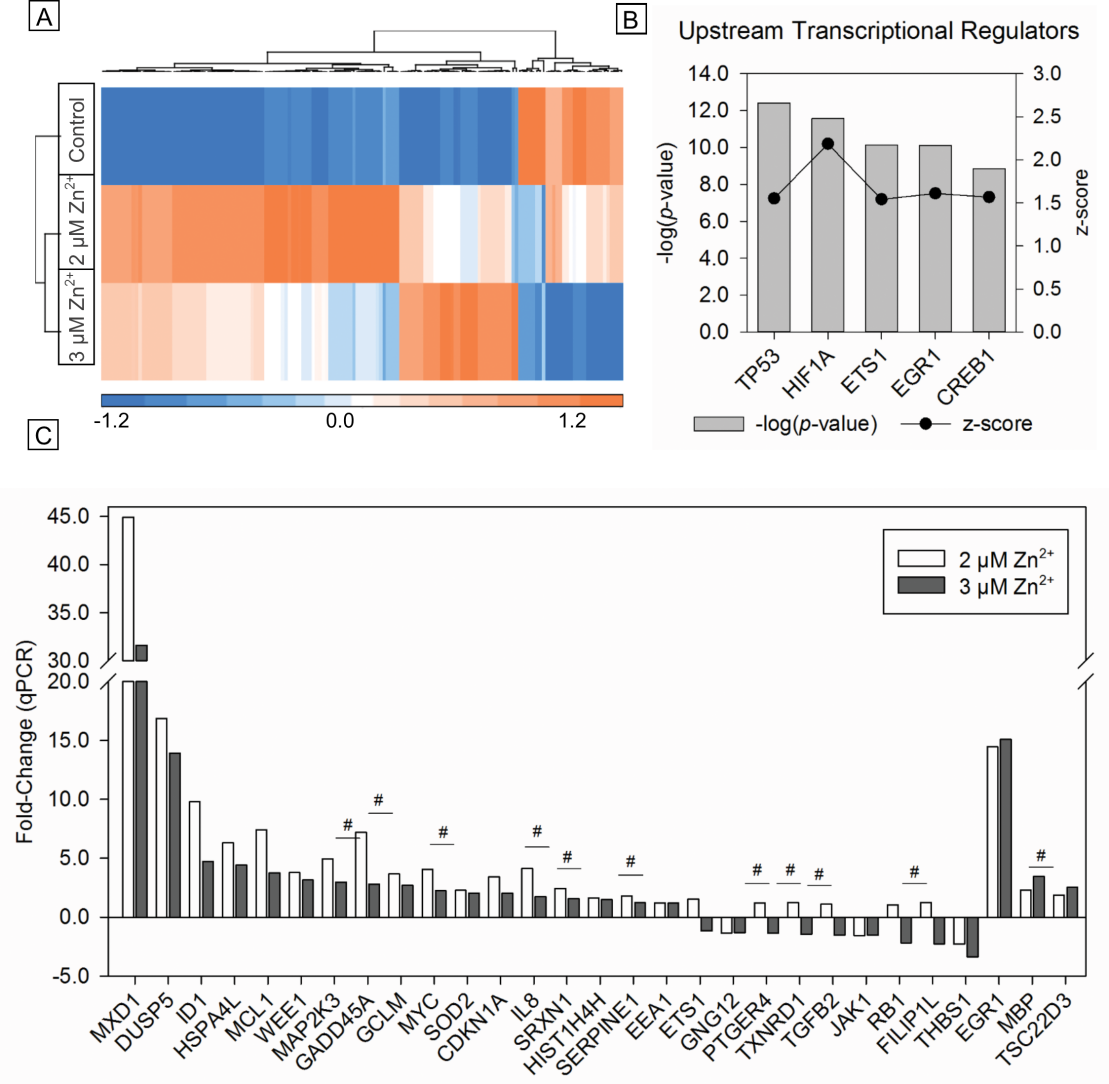
Biomarker gene set delineating adaptive and adverse response in Zn^2+^-exposed BEAS-2B cells. (A) Unsupervised hierarchical clustering of the 154 significantly differentially expressed gene probes between the adaptive (2 μM) and cytotoxic (3 μM) Zn^2+^ exposures after 4 h by two sample t-test. Log_2_ normalized probe intensity values are shifted to a mean of zero and scaled to a standard deviation of 1. (B) Analysis of upstream transcriptional regulators by Ingenuity Pathway Analysis is presented as –log(*p*-values) based on overlap of biomarker genes and known transcription factor targets by Fisher’s exact test. The z-score infers the activation state of the transcription factor based on the observed gene regulation. (C) Validation of the biomarker gene set in an independent experiment by RT-qPCR. Fold-change compared with control were calculated using *HMBS* and *ACTB* normalized values (n = 4). Significant difference (*p* <0.05) compared with each other (#) by two sample t-test.

We further analyzed early gene expression changes by comparing probe intensities of the 794 DEGs at 4 h after adaptive (2 μM Zn^2+^) or cytotoxic (3 μM Zn^2+^) exposures using two-sample t-test and found 154 genes significantly differed between the two exposures (S1 Table). Hierarchical clustering of these biomarker genes indicated clear differences in the levels of gene expression between the adaptive and cytotoxic doses (Fig 5A). Interestingly, all but one of the 54 genes differentially expressed in the adaptive dose were upregulated versus control, whereas roughly half of the 50 genes differentially regulated in only the cytotoxic dose were upregulated. Moreover, 32% of the biomarker genes derived by two-sample t-test were significantly up- or down-regulated under both exposure conditions suggesting that magnitude of expression is driving the apical, downstream cellular responses. Upstream regulator analysis revealed numerous transcription factors associated with the expression changes of the biomarker genes. The top five significantly regulated transcription factors included p53, HIF1A, ETS1, EGR1, and CREB, which exhibited putative activation based on downstream gene regulation (Fig 5B). We associated 36 of the 154 biomarker genes with the p53 pathway. Using a database of p53 ChIP-seq studies (see material and methods), we linked 17 of these 36 genes, plus an additional 23 biomarker genes, as likely direct targets of p53. Gene pathway analysis demonstrated significant enrichment of numerous canonical signaling pathways associated with cytotoxicity, cell death, and oxidative stress response (S2 Table). Furthermore, *NRF2-mediated Oxidative Stress Response* and *p53 Signaling* were among the top significantly enriched toxicity pathways (S3 Table).

In an independent experiment, we investigated 29 genes from the biomarker set by RT-qPCR to confirm the Zn^2+^-mediated changes after 4 h of exposure (Fig 5C). Tabular results of this comparison are presented in S4 Table. Of the 29 biomarker genes investigated by RT-qPCR, 11 exhibited significant expression differences between the 2 μM and 3 μM Zn^2+^ exposure groups by two-sample t test (*p* <0.05). Furthermore, 11 additional targeted transcripts exhibited consistent differences compared with the microarray analysis but did not reach statistical significance resulting in a general concordance for 76% (22/29) of probed transcripts.

Next, we utilized a commercially available database to compare the biomarker set to other publically available gene expression biosets to investigate its relevance to additional exposures. The top 20 positively correlated biosets of bronchial origin for each Zn^2+^ exposure, calculated with a Running Fisher test, were merged and are presented in Table 1. The most highly significant biosets, -log(*p*-values), ranging from 6.4 to 14.37 originated from cigarette and tobacco smoke exposures with 51–68 genes positively correlated with our biomarker set. Exposure to viral components, bacteria, inflammatory agents, a polycyclic aromatic hydrocarbon, and mechanical injury also resulted in significant correlation to our biomarker. Notably, all top-ranked biosets were more highly significant under adaptive Zn^2+^ exposure conditions except in normal human bronchial epithelial (NHBE) cells 24 h after 15 minute cigarette exposure.

**Table 1:** Comparison of the biomarker to bronchial gene expression biosets in NextBio^a^.

					2 μM Zn^2+^ (adaptive)		3 μM Zn^2+^ (cytotoxic)	
Public ID	Cell Type	Exposure/ Condition	Recovery	Control	+ Correlated Genes	-log(*p*-value)	+ Correlated Genes	-log(*p*-value)
GSE10718	NHBE	15 min cigarette smoke	2 h	Air	56	14.37	56	13.32
		15 min cigarette smoke	4 h	Air	62	11.96	62	11.30
		15 min cigarette smoke	24 h	Air	53	7.08	54	7.66
GSE10700	NHBE	15 min light cigarette smoke	4 h	Mock	54	13.38	54	11.70
		15 min reference cigarette smoke	4 h	Mock	51	10.35	51	8.82
E-TABM-127	NHBE	1 h tobacco smoke	23 h	Untreated	61	13.15	59	11.11
		1 h tobacco smoke	5 h	Untreated	55	12.35	55	11.27
		1 h tobacco smoke	23 h	Air	68	11.32	65	10.22
		1 h air	5 h	Untreated	37	7.72	35	6.41
		1 h air	-	Untreated	28	7.70	28	7.24
GSE6802	BEAS-2B	4 h UV-inactivated RSV	-	Untreated	53	13.01	53	12.72
GSE59128	AEC	Mechanical Injury + Cyclic Stretch	8 h	Sham	31	12.36	29	10.64
	dAEC	Mechanical Injury	8 h	0 h	40	8.21	39	7.15
GSE47460	Lung Tissue	Stage 2 COPD	-	Stage 1	26	9.96	25	8.49
		Stage 4 COPD	-	Stage 1	39	9.13	38	8.60
E-MTAB-874	AIR-100	28 min 15% cigarette smoke	24 h	Air	56	9.52	54	8.02
GSE65018	S9	2 h 2000ng/mL staphylococcal Hla		Mock	21	8.54	21	8.24
GSE16650	BEAS-2B	4hr mechanical stretch + 20 ng/mL TNF-α		Untreated	32	8.44	31	7.49
GSE34635	BEAS-2B	24 h 1 μM N-OH-PhIP	-	DMSO	85	7.68	85	7.18
GSE19392	NHBE	18h supernatant of RBCs bound to PR8-dNS1	-	Untreated	40	7.40	40	7.41
		18h supernatant of RBCs bound to PR8	-	Untreated	36	7.35	37	7.27

^a^The biomarker was imported into NextBio, genes were rank-ordered based on their fold-change. The biomarker was then compared to publically available biosets in the NextBio database using a pair-wise rank-based algorithm (the Running Fisher test) and then filtered for biosets of human bronchial origin [31].

**Fig 6:**
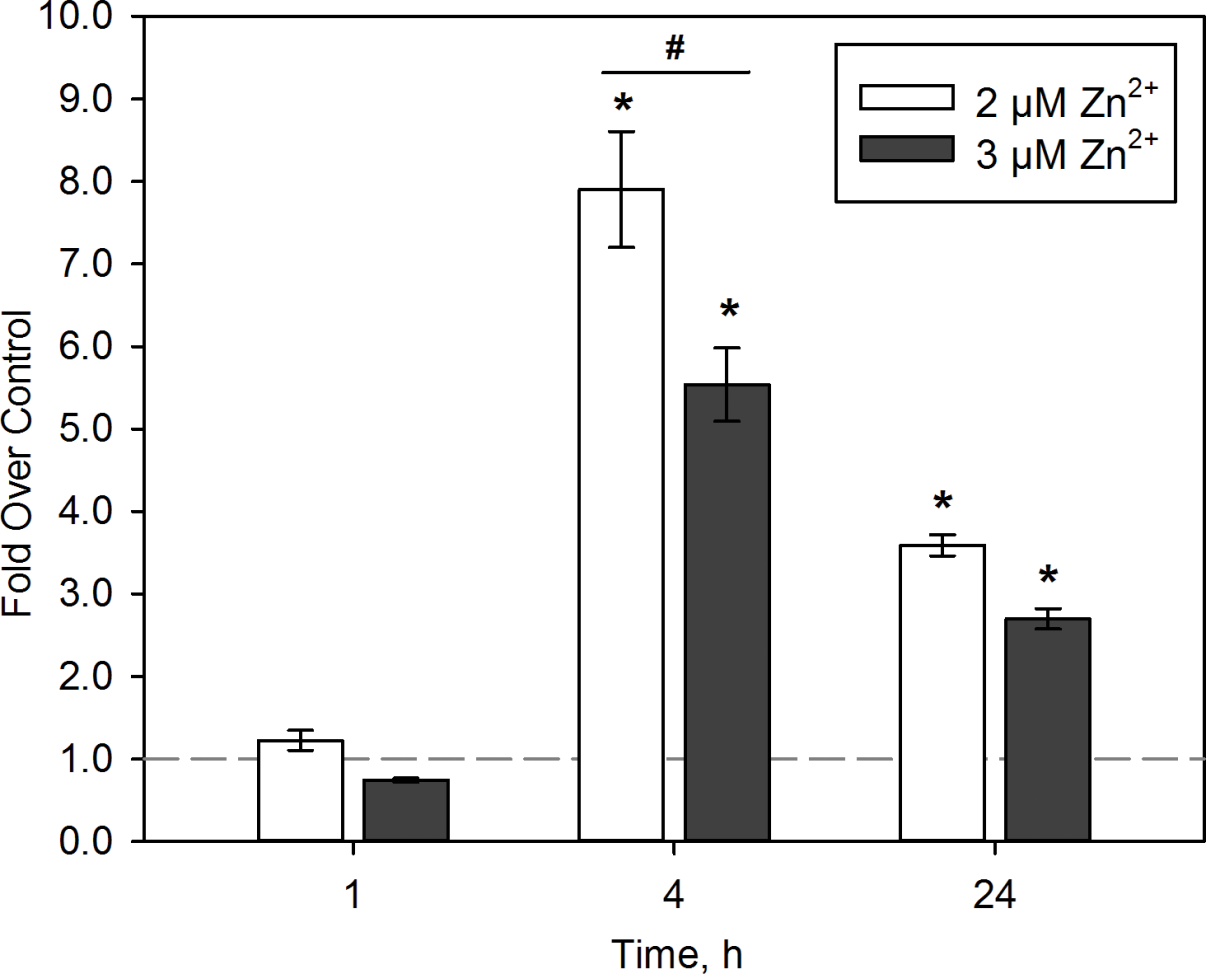
Early Zn^2+^-mediated oxidative stress in BEAS-2B cells. Analysis of activated NRF2 protein in nuclear extracts of BEAS-2B cells exposed to 2 and 3 μM Zn^2+^ in the presence of 1 μM pyrithione for up to 24 h. Each bar represents the fold change over unexposed control wells (dotted line) measured by an ELISA-based sequence binding assay. (*n* = 3, mean ± SD). Significance (*p* <0.01) compared with control (*) or each other (#) by one-way ANOVA with Holm-Sidak posttest.

Because Zn^2+^ exposure is extensively linked to oxidative stress and p53 potentiates NRF2 signaling through its target gene, p21 [42], we examined the biomarker for enrichment of NRF2-related genes. Of the 154 biomarker genes, 20 were related to NRF2 signaling, 10 of which overlap with the p53 pathway. Furthermore, utilizing previously published NRF2 ChIP-seq and gene expression data, half of the NRF2-related genes were identified as likely direct transcriptional targets [40]. Notably, all NRF2 related genes were more highly expressed under adaptive conditions with the exception of *HISH1H4H* and *SOD2*. To confirm an early oxidative stress response and investigate drivers of the magnitude expression changes observed in the biomarker genes, we measured activated NRF2 in the nuclear extracts of BEAS-2B cells exposed to 2 and 3 μM Zn^2+^ in the presence of 1 μM pyrithione for 1, 4, and 24 h (Fig 6). No significant changes in NRF2 activation were observed in BEAS-2B cells after 1 h exposure, however, 2 and 3 μM Zn^2+^ exposure exhibited 7.9±0.7 and 5.5±0.4 fold activation over control, respectively. Interestingly, 2 μM Zn^2+^ elicited significantly more NRF2 activation compared with 3 μM Zn^2+^, which is consistent with the higher expression of NRF2-related genes by microarray analysis. After 24 h, activation reduced to 3.6±0.1 and 2.7±0.1 fold over control in cells exposed to 2 and 3 μM Zn^2+^, respectively. Total (GSH) and oxidized (GSSG) glutathione levels were then measured to confirm alterations in redox state after Zn^2+^ exposure. Total GSH levels were not significantly altered with Zn^2+^ exposure. However, GSSG was significantly increased from 0.08±0.02 μM in control cells to 0.16±0.04 μM and 0.18±0.01 μM in 2 and 3 μM Zn^2+^-exposed cells, respectively, resulting in significantly decreased GSH/GSSG ratios (S4 Fig). No significant difference was observed between control and pyrithione only exposed cells.

## Discussion

Considerable effort has focused on utilizing stress response pathways in HTS paradigms and risk assessment frameworks to rapidly prioritize and characterize hazard associated with environmental exposures [3, 7, 43, 44]. However, studies identifying mechanism-based biomarkers that distinguish adverse from adaptive cellular processes are limited. In our study, we characterized a point of departure for cytotoxic response in a model of oxidant exposure in human airway cells. We then utilized whole genome transcriptomics and identified early dampening of adaptive response genes under cytotoxic exposure conditions prior to the appearance of typical apical endpoints.

Cell viability studies indicate a point of departure for cytotoxic responses at exposures between 2 and 3 μM Zn^2+^. Although our initial efforts to confirm the mechanism of cytotoxicity were unclear, we observed significant decreases in the anti-apoptotic markers Mcl-1/Bak and survivin with ≥ 2.5 μM Zn^2+^exposure. Here, reduced levels indicate activation of canonical apoptotic signaling. In contrast, Zn^2+^ exposure resulted in increased levels of Bcl-xL/Bak, another anti-apoptotic marker. Jung et al. 2002 reported Zn^2+^-mediated induction of Bcl-xL, which could in turn increase levels of the Bcl-xL/Bak complex [45]. Because both Bcl-xL/Bak and Mcl-1/Bak prevent Bak oligomerization in the mitochondrial outer member, reduced levels of one complex could potentiate Bak-mediated apoptotic signaling. Zn^2+^ exposure has also been shown to inhibit caspase-3 cleavage [46, 47], and in agreement, we found that cleaved caspase-3 decreased in a time- and concentration-dependent manner with Zn^2+^ exposure. Overall, we observed alterations in apoptotic signaling markers, and results from longer exposures likely reflect events downstream of initial apoptotic signaling that ultimately results in the reduction of cell viability evident after 24 h exposure to 3 μM Zn^2+^.

Activation of p53 through posttranslational modifications mediates several cellular responses, including cell cycle arrest, senescence, and apoptosis [48]. We observed significant increases in p53 phosphorylation at Ser 20 after 4 h exposure to both 2 and 3 μM Zn^2+^. In addition, Zn^2+^ exposure elicited slight increases in the levels of p53 phosphorylated at Ser 15 and the pro-apoptotic marker, cleaved PARP. These results clearly indicate activation of p53; however, there were no significant differences in activation between the adaptive (2 μM) and cytotoxic (3 μM) Zn^2+^ exposures after 4 h. While the activation profile of p53 is similar between exposures, other transcription cofactors or post-translational modifications may mediate the observed differences in p53 target genes [49–53]. Interestingly, wild-type p53 protein was significantly decreased after Zn^2+^ exposure. The BEAS-2B cell line is SV40 transformed, in which the large T antigen binds to p53, preventing the activation of a p53-mediated transcriptional response that contributes to the limited proliferative abilities of primary cells [54]. Despite the interaction of the large T antigen with p53, numerous environmentally relevant exposures, including metals, have been shown to activate p53 and downstream targets in BEAS-2B cell cultures [55–57]. Moreover, the p53 gene in the BEAS-2B cells line contains two germline missense mutations in codons 47 and 72; however, the p53 protein exhibits normal wild type properties [58]. While the characteristics of p53 in this SV40 transformed cell line may pose a limitation for direct comparison with normal human bronchial epithelial cells, our results provide a clear delineation of adaptive and adverse responses related to p53 activation, which merit further investigation. Overall, studies assessing the Zn^2+^-mediated cytotoxicity and activation of p53 support a tipping point from adaptive, recoverable cellular processes to an unrecoverable, cytotoxic response with exposures ≥2.5 μM Zn^2+^.

To our knowledge, this study is the first to develop an early gene expression signature delineating adaptive from adverse oxidative effects in human lung cells. Our biomarker includes 154 genes that are significantly differentially expressed between adaptive and cytotoxic exposure conditions. When comparing gene expression after adaptive exposure relative to cytotoxic conditions, we observed enrichment of several stress-related signaling pathways and upstream transcription factor regulation. Interestingly, the top toxicity pathways included NRF2-mediated oxidative stress response and p53 signaling (S3 Table). Here, canonical p53 signaling contributes to a limited set of adaptive and apoptotic cellular responses, requiring further analysis of the genes expressed in this pathway to identify the underlying mechanisms of these responses. Upstream regulator analysis revealed p53 as the most highly enriched transcription factor (Fig 5B). In agreement with canonical and toxicity pathway analysis, NRF2 was also significant with a -log(*p*-value) of 3.0 (data not shown). While considerable evidence implicates crosstalk between p53 and NRF2 pathways as coordinating the p53-initiated cell survival response [42, 59], less is known about the molecular mechanisms responsible for the shift to programmed cell death. Increased activation of NRF2 was present at the adaptive, 2 μM Zn^2+^ exposure compared with the cytotoxic 3 μM Zn^2+^ exposure (Fig 6). *CDKNA1* (p21), a p53 target gene critical for cell survival through the initiation of cell cycle arrest, has been shown to stabilize and prevent degradation of NRF2 [42]. In agreement, *CDKNA1*, a biomarker gene, is more highly upregulated in our model under adaptive conditions by microarray and qPCR analysis after 4 h, which may account for the increase NRF2 activation under adaptive Zn^2+^ exposure conditions (S1 Table).

It has been reported that p53 can repress transcription of NRF2 target genes, including *SCL7A11*, *NQO1*, and *GSTA1* [59, 60]. While genes previously shown to be targets of p53-mediated down-regulation were not significantly altered in our model, all NRF2 related genes in the biomarker set were expressed at lower levels under cytotoxic conditions with the exception of *HISH1H4H* and *SOD2*. These findings further support the magnitude of response and contribution of NRF2 as mediating factors in the transition between adaptive and adverse cellular responses. Additionally, Chen et al. 2012 reported the biphasic regulation of NRF2 by p53 expression [59]. Thus, at low p53 levels, NRF2 is more highly expressed; while at higher p53 levels, NRF2 is repressed. This suggests that crosstalk, either at the DNA binding level or upstream signaling, is key for p53-mediated tumor suppression through coordinating the pathways critical for cell survival and death. In our studies, the exact mechanism of the early magnitude expression differences between adaptive and cytotoxic exposures is unclear, highlighting the necessity for further investigations.

We utilized Zn^2+^ exposures to generate oxidative stress in a reproducible manner. This environmentally relevant exposure model may apply to other metal exposures, including cadmium [61] and mercury [62], which can displace protein bound Zn, thereby increasing intracellular free Zn^2+^ and adaptive and adverse cellular effects [63, 64]. Moreover, the enrichment of our biomarker gene set in other publicly available gene expression data of bronchial origin suggests a similar biological response, likely oxidant-mediated, with other environmental exposures and related diseases, including cigarette smoke, bacterial or viral infection, mechanical injury, COPD, and PAHs (Table 1). Our approach could be expanded to assess adverse and adaptive responses due to multiple contaminants or complex chemical mixtures that more accurately represent environmental exposures.

Taken together, our findings suggest that the switch from stress adaptation and cytotoxicity in our model begins to occur at exposures of approximately 2 μM Zn^2+^, and as early as 4 h after exposure, based on gene expression changes. These alterations are measurable earlier than typically measured phenotypic endpoints of apoptosis. Additional analysis of p53 and NRF2-related genes in this transcriptomic biomarker reveals dampening of an adaptive response compared to cytotoxic Zn^2+^ exposures. Future work will determine molecular mechanism responsible for these magnitude gene expression changes. Our findings will help us ultimately identify early and measurable points of departure for adverse (versus simply adaptive) events *in vitro*. These assessments will improve high-throughput *in vitro* and *in silico*-based screening models for predicting adverse effects on human health due to chemical and environmental-based exposure.

## Supporting Information

S1 Fig Time- and concentration-dependent cytotoxicity after Zn^2+^ exposure in BEAS-2B cells. MTT assay was used to measure cytotoxicity in BEAS-2B cells exposed to 0–10 μM Zn^2+^ and 1 μM pyrithione for up to 40 h. Results are presented as percent of unexposed control wells (*n* = 3, mean ± SD). (*) *p* <0.01 compared with control by one-way ANOVA followed by Holm-Sidak post hoc test.
(PDF)

S2 Fig Unique and overlapping differentially expressed genes after Zn^2+^ exposure in BEAS-2B cells. Venn diagrams depicting the number of DEGs uniquely or similarly expressed in the indicated exposure groups by all Zn^2+^ concentration (top) or by duration (bottom).
(PNG)

S3 Fig Zn^2+^-mediated signaling pathway enrichment in BEAS-2B cells. Ingenuity Pathway Analysis was used to merge the top three canonical pathways represented by genes expressed in each treatment group. A heat map of enrichment scores quantified as -log(*p*-value) are displayed. Significant enrichment is considered at -log(*p*-value) ≥1.3, which corresponds to *p* <0.05.
(PDF)

S4 Fig Early Zn^2+^-mediated oxidative stress in BEAS-2B cells. The GSH/GSSG ratio was measured in whole cell lysates exposed to up to 3 μM Zn^2+^ in the presence of 1 μM pyrithione for 2 h by luciferase-based reporter assay (*n* = 3, mean ± SD). Significance (*p* <0.01) compared with control (*) or each other (#) by one-way ANOVA with Holm-Sidak posttest.
(PDF)

S1 Table Genes delineating early adaptive and cytotoxic responses after 4 h Zn^2+^ exposure (XLSX)

S2 Table Ingenuity Pathway Analysis (IPA) was used determine significant enrichment of canonical signaling pathways represented by the 154 biomarker genes. (XLSX)

S3 Table Ingenuity Pathway Analysis (IPA) was used determine significant enrichment of toxicity pathways represented by the 154 biomarker genes. (XLSX)

S4 Table RT-qPCR validation of microarray analysis and biomarker selection. (XLSX)

## Abbreviations

AEC: primary human airway epithelial cells
AIR-100: differentiated normal human bronchial epithelial cells
ARE: antioxidant response element
BEAS-2B: an SV-40 transformed human bronchial epithelial cell line
CNRQ: calibrated normalized relative quantities
COPD: chronic obstructive pulmonary disease
dAEC: differentiated human airway epithelial cells
DEG: differentially expressed gene
GSH: reduced glutathione
GSSG: oxidized glutathione
HCI: high-content imaging
Hla: alpha-toxin
HTS: high-throughput screening
KBM: keratinocyte basal medium
KGM: keratinocyte growth medium
MTT: 3-(4,5-dimethylthiazol-2-yl)-2,5-diphenyltetrazolium bromide
NFE2L2 or NRF2: nuclear factor erythroid 2-like 2
NHBE: normal human bronchial epithelial
N-OH-PhIP: N-hydroxy-2-amino-1-methyl-6-phenylimidazo[4,5-b]pyridine
PR8: a wild-type influenza virus
PR8-dNS1: a mutant influenza virus
RSV: respiratory syncytial virus
RT-qPCR: quantitative real-time polymerase chain reaction
S9: a human bronchial epithelial cell line
TNF-α: tumor necrosis factor alpha
UV: ultraviolet
Zn: zinc
