# Supplementary figures and images for: Developing a Gene Biomarker at the Tipping Point of Adaptive and Adverse Responses in Human Bronchial Epithelial Cells
Source: PLoS One. 2016 May 19;11(5):e0155875. doi: 10.1371/journal.pone.0155875 (PMC4873291; doi:10.1371/journal.pone.0155875)

% of Control

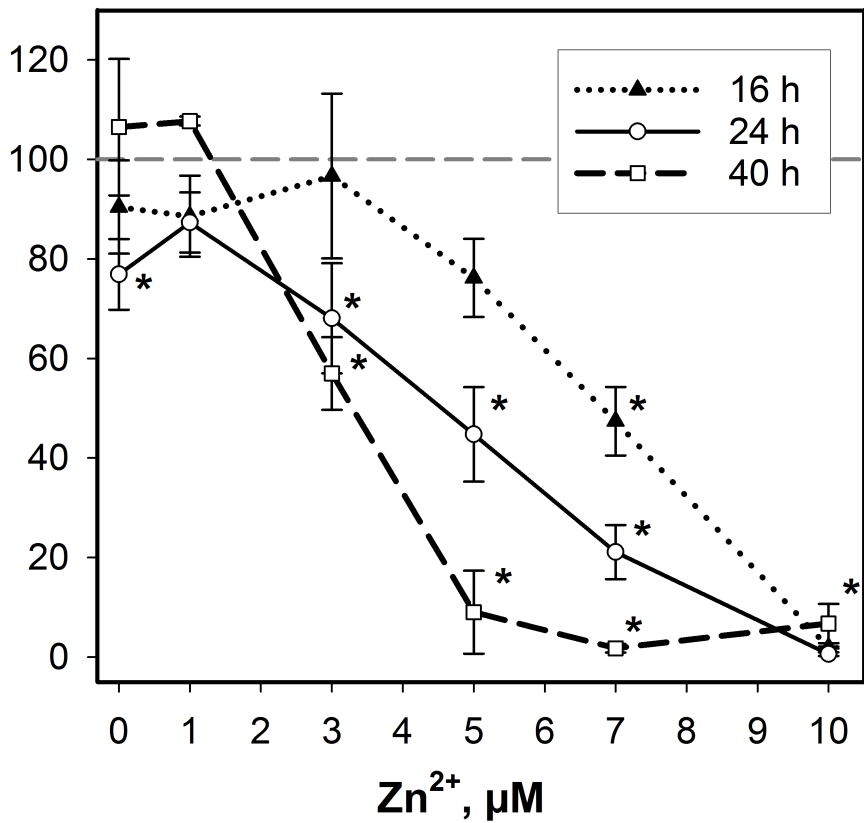

Supplement: S1 Fig — MTT assay was used to measure cytotoxicity in BEAS-2B cells exposed to 0–10 μM Zn2+ and 1 μM pyrithione for up to 40 h. Results are presented as percent of unexposed control wells (n = 3, mean ± SD). (*) p <0.01 compared with control by one-way ANOVA followed by Holm-Sidak post hoc test. (PDF) [file pone.0155875.s001.pdf]

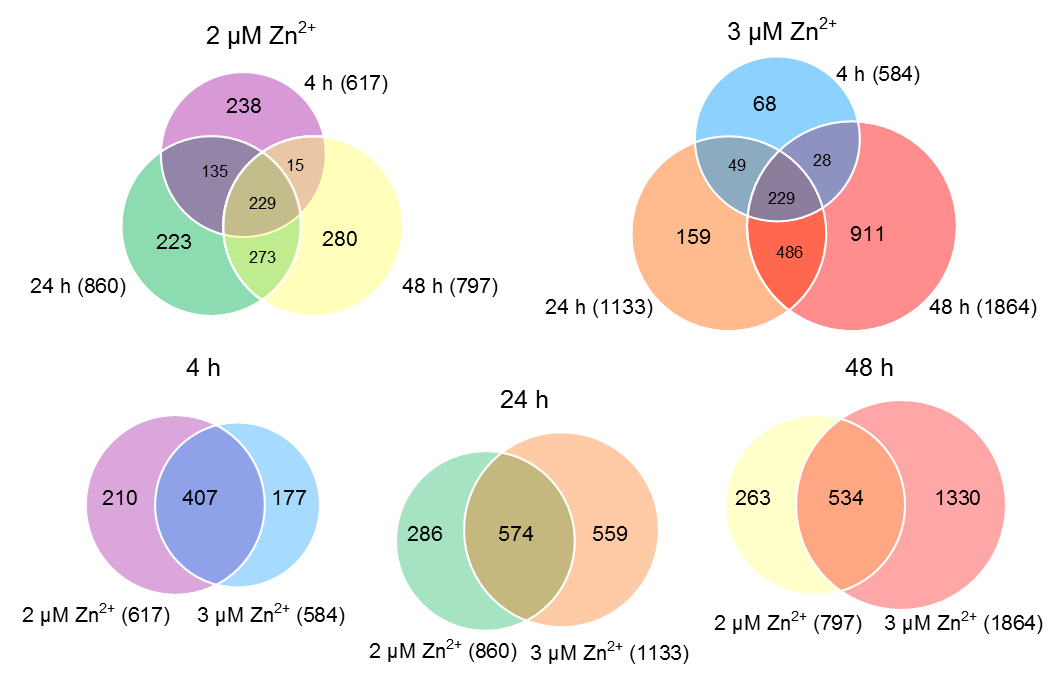

Supplement: S2 Fig — Venn diagrams depicting the number of DEGs uniquely or similarly expressed in the indicated exposure groups by all Zn2+ concentration (top) or by duration (bottom). (PNG) [file pone.0155875.s002.png]

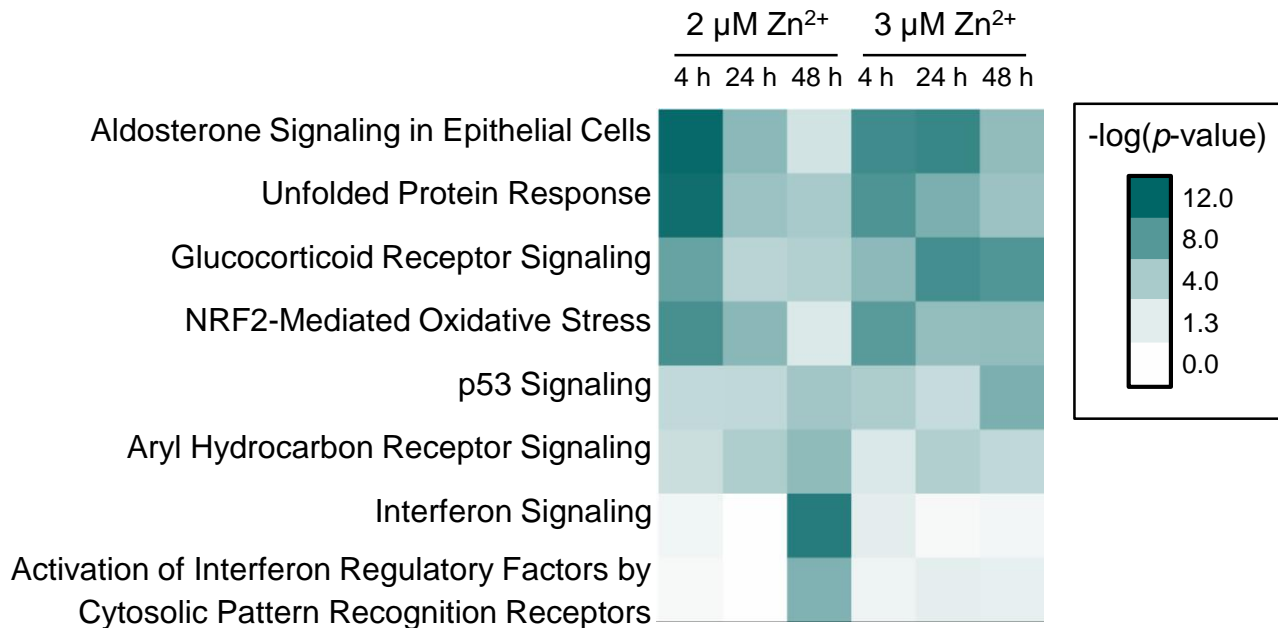

Supplement: S3 Fig — Ingenuity Pathway Analysis was used to merge the top three canonical pathways represented by genes expressed in each treatment group. A heat map of enrichment scores quantified as -log(p-value) are displayed. Significant enrichment is considered at -log(p-value) ≥1.3, which corresponds to p <0.05. (PDF) [file pone.0155875.s003.pdf]

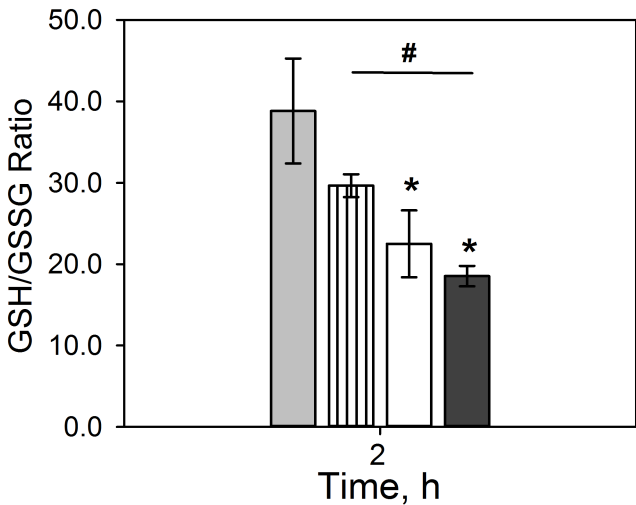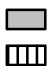

Control

0  $\mu\text{M}$   $\text{Zn}^{2+}$

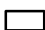

2  $\mu\text{M}$   $\text{Zn}^{2+}$

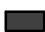

3  $\mu\text{M}$   $\text{Zn}^{2+}$

Supplement: S4 Fig — The GSH/GSSG ratio was measured in whole cell lysates exposed to up to 3 μM Zn2+ in the presence of 1 μM pyrithione for 2 h by luciferase-based reporter assay (n = 3, mean ± SD). Significance (p <0.01) compared with control (*) or each other (#) by one-way ANOVA with Holm-Sidak posttest. (PDF) [file pone.0155875.s004.pdf]
